# Diffuse leptomeningeal glioneuronal tumor: a double misnomer? A report of two cases

**DOI:** 10.1186/s40478-020-00978-7

**Published:** 2020-06-30

**Authors:** Romain Appay, Mélanie Pages, Carole Colin, David T. W. Jones, Pascale Varlet, Dominique Figarella-Branger

**Affiliations:** 1 APHM, CHU Timone, Service d’Anatomie Pathologique et de Neuropathologie, Marseille, France; 2 Aix-Marseille Univ, CNRS, INP, Inst Neurophysiopathol, Marseille, France; 3 Université Paris Descartes, Sorbonne Paris Cité, Paris, France; 4 Service de Neuropathologie, GHU Paris – Hôpital Sainte-Anne, Paris, France; 5 Equipe SiRIC RTOP Recherche Translationelle en Oncologie Pédiatrique, Institut Curie, Paris, France; 6 INSERM U830, Laboratoire de Génétique et Biologie des Cancers, Institut Curie, Paris, France; 7 SIREDO: Care, Innovation and Research for Children, Adolescents and Young Adults with Cancer, Institut Curie, Paris, France; 8 Hopp Children’s Cancer Center Heidelberg (KiTZ), 69120 Heidelberg, Germany; 9 Pediatric Glioma Research Group, German Cancer Research Center (DKFZ), Im Neuenheimer Feld 280, 69120 Heidelberg, Germany; 10 INSERM UMR 1266, IMA-Brain, Institut de Psychiatrie et de Neurosciences de Paris, Paris, France

**Keywords:** DLGNT, Adult, Supratentorial, *BRAFV600E* mutation, DNA-methylation profiling

## Abstract

Diffuse leptomeningeal glioneuronal tumor (DLGNT) was introduced, for the first time, as a provisional entity in the 2016 WHO classification of central nervous system tumors. DLGNT mainly occur in children and characterized by a widespread leptomeningeal growth occasionally associated with intraspinal tumor nodules, an oligodendroglial-like cytology, glioneuronal differentiation and MAP-Kinase activation associated with either solitary 1p deletion or 1p/19q codeletion in the absence of *IDH* mutation.

We report here two unexpected DLGNTs adult cases, characterized by a unique supratentorial circumscribed intraparenchymal tumor without leptomeningeal involvement in spite of long follow-up. In both cases, the diagnosis of DLGNT was made after DNA-methylation profiling which demonstrated that one case belonged to the DLGNT class whereas the other remained not classifiable but showed on CNV the characteristic genetic findings recorded in DLGNT. Both cases harbored 1p/19q codeletion associated with *KIAA1549:BRAF* fusion in one case and with *BRAF V600E* and *PIK3CA E545A* mutations, in the other.

Our study enlarges the clinical and molecular spectrum of DLGNTs, and points out that the terminology of DLGNTs is not fully appropriate since some cases could have neither diffuse growth nor leptomeningeal dissemination. This suggests that DLGNTs encompass a wide spectrum of tumors that has yet to be fully clarified.

## Introduction

Diffuse leptomeningeal glioneuronal tumor (DLGNT) was introduced as a provisional entity in the 2016 WHO classification of tumors of the central nervous system (CNS) [1]. It was defined as *« a rare glioneuronal neoplasm characterized by predominant and widespread leptomeningeal growth, an oligodendroglial-like cytology, evidence of neuronal differentiation in a subset of cases, and a high rate of concurrent KIAA1549:BRAF gene fusions and either solitary 1p deletion or 1p/19q codeletion in the absence of IDH mutation ».* DLGNT has not yet been assigned a WHO grade, due to the limited number of reported cases, the often incomplete clinical follow-up, and the variability of outcome. DLGNT mainly occur in children and young adults. In addition to the leptomeningeal growth, more circumscribed, intraparenchymal, small cystic or solid tumor nodules may be observed. These tumor nodules are usually intraspinal but could be intracerebral in rare cases [2–5]. However, recent studies have reported DLGNT cases without diffuse growth nor leptomeningeal dissemination, at diagnosis and during long follow-up, suggesting that DLGNTs do not necessarily present with leptomeningeal dissemination on MRI [6–8]. Accordingly, DLGNTs were defined in the sixth cIMPACT-NOW update as “A glioneuronal neoplasm composed of oligodendrocyte-like cells; chromosome arm 1p deletion and a mitogen-activated protein kinase (MAP-Kinase) pathway gene alteration, *KIAA1549:BRAF* fusion being most frequent; without *IDH* mutation; and commonly with diffuse leptomeningeal tumor spread” [9]. Because the pathological features of DLGNT are not specific, and with a large panel of differential diagnoses including pilocytic astrocytoma, ganglioglioma or extraventricular neurocytoma, the diagnosis might be particularly challenging in the cases lacking leptomeningeal dissemination. In a recent paper, Deng et al. [4] have reported the usefulness of DNA-methylation profiling for the diagnosis of DLGNTs. Copy-number profiles derived from the methylation arrays revealed unifying characteristics, including loss of chromosomal arm 1p in all cases. Furthermore, the authors showed that the molecular DLGNT class can be subdivided into two subgroups with distinct molecular alterations and clinical course. According to these findings, the c-IMPACT-NOW consortium suggested that DLGNT should be considered as a distinct tumor type with two distinct subtypes recognized on DNA-methylation profiling [9].

We report here two very unusual cases of DLGNT occurring in young adults and both presenting as a unique intracerebral mass without leptomeningeal involvement in spite of long follow-up.

## Case presentation

### Case 1

**Fig. 1:**
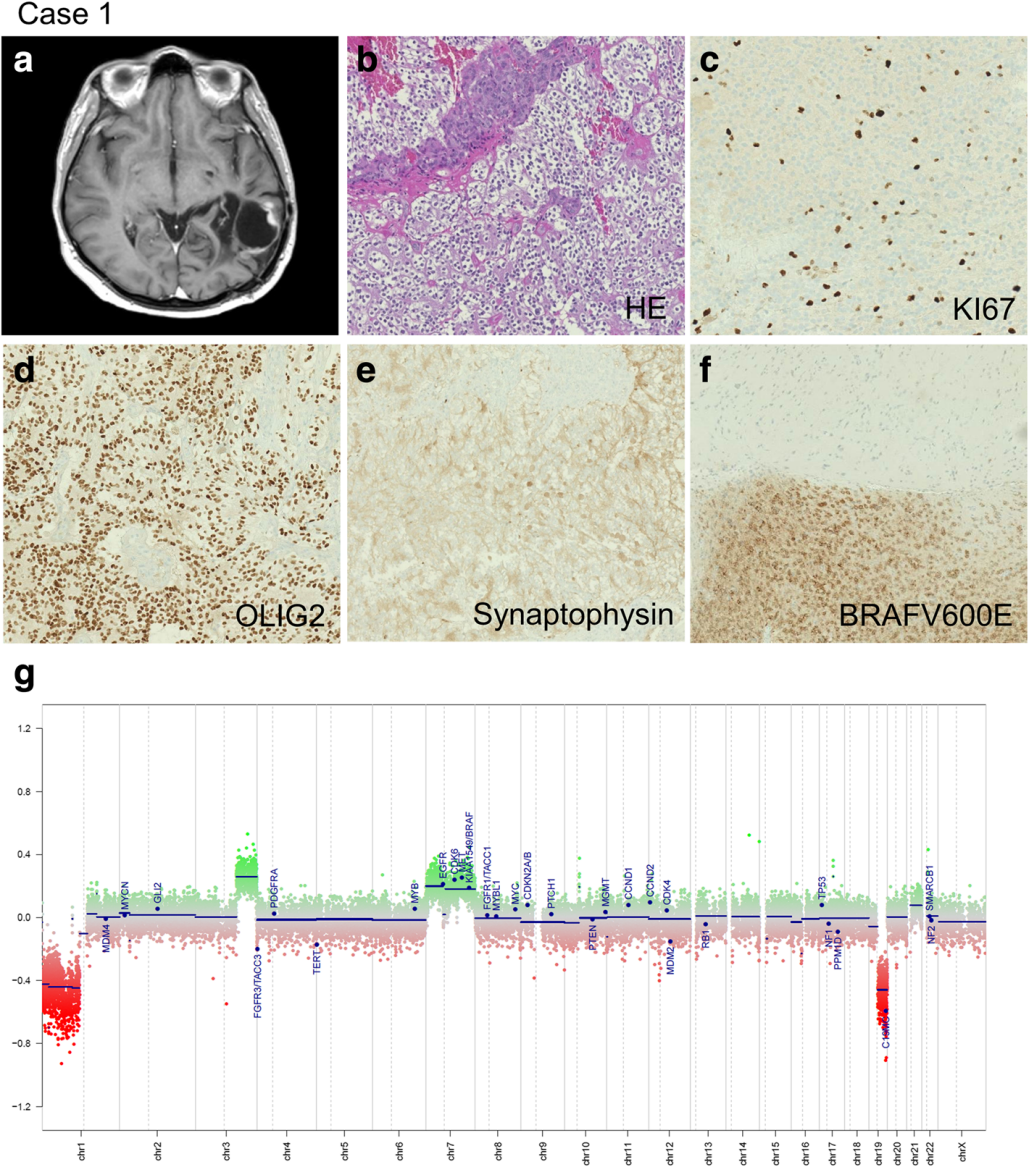
**a** MRI disclosed a nodule enhanced after gadolinium injection, located within the intraoperative cavity. **b** High cellularity neoplasm composed of relatively monomorphic oligodendrocyte-like cells associated with endocrinoïd vasculature. **c** KI67 labeling index was around 10%. **d**, **e**, **f** Tumor cells strongly expressed OLIG2 and synaptophysin and BRAFV600E. **g** Copy-number profile derived from DNA-methylation arrays revealed 1p/19q codeletion with additional gains of chromosomes 3 and 7

A 35-year-old woman was admitted to La Timone Hospital for epileptic seizures. She had history of a left parietal mass that was resected and diagnosed in Armenia as “diffuse astrocytoma grade II” when she was 14, without postoperative treatment. Unfortunately, we could not retrieve the tumor sample in order to review the initial diagnosis. MRI revealed an enhanced nodule after gadolinium injection, arising within the previous resection cavity (Fig. 1, a) without leptomeningeal involvement. Karnofsky index was 90 and no neurological deficit was recorded although her verbal fluency was decreased. After 2 years of MRI and clinical follow-up, a slight increase in the nodule size was observed, which, in spite of normal FDG-PET uptake, motivated a surgical excision and a gross total resection was achieved.

Pathological examination showed a highly cellular neoplasm composed of relatively monomorphic oligodendrocyte-like cells associated with an endocrinoïd vasculature. Microvascular proliferation was observed without necrosis (Fig. 1, b). Mitotic activity was high with up to 6 mitotic figures per 2.4 mm^2^. KI67 labeling index was around 10% (Fig. 1, c). Tumor cells strongly expressed OLIG2 and to a lesser extent synaptophysin but not GFAP nor CD34 (Fig. 1, d-e). IDH1R132H was negative and ATRX expression was retained in nuclei. BRAF V600E expression (VE1 antibody) was strong in all tumor cells (Fig. 1, f).

Targeted NGS analysis confirmed the *BRAF V600E* mutation and revealed a co-occurrence of the hotspot mutation *PIK3CA* E545A. No *IDH*, *H3F3A/HIST1H3B* nor *TERT* promoter mutation were observed. FISH analysis showed a 1p/19q codeletion. Diagnosis was “unusual glioneuronal tumor with histological features of anaplasia”. Central review was performed under the framework of the national RENOCLIP-LOC network and DNA-methylation profiling was performed using an EPIC array (Integragen, Evry, France) as previously described [10]. The raw IDAT files were uploaded to https://www.molecularneuropathology.org for supervised analysis using the Random Forest methylation class prediction algorithm, and copy number profiles were obtained as previously described [11]. The methylation class was DLGNT (calibrated score 0.99) and CNV analysis confirmed 1p/19q codeletion and showed additional gains of chromosomes 3 and 7 (Fig. 1, g). Comparison with the DLGNT reference series indicated that this tumor belongs to DLGNT – molecular class 1. Final diagnosis was recurrent “localized form of DLGNT associated with 1p/19q codeletion and *BRAF V600E* mutation”. It was decided to closely follow the patient without adjuvant treatment.

### Case 2

**Fig. 2:**
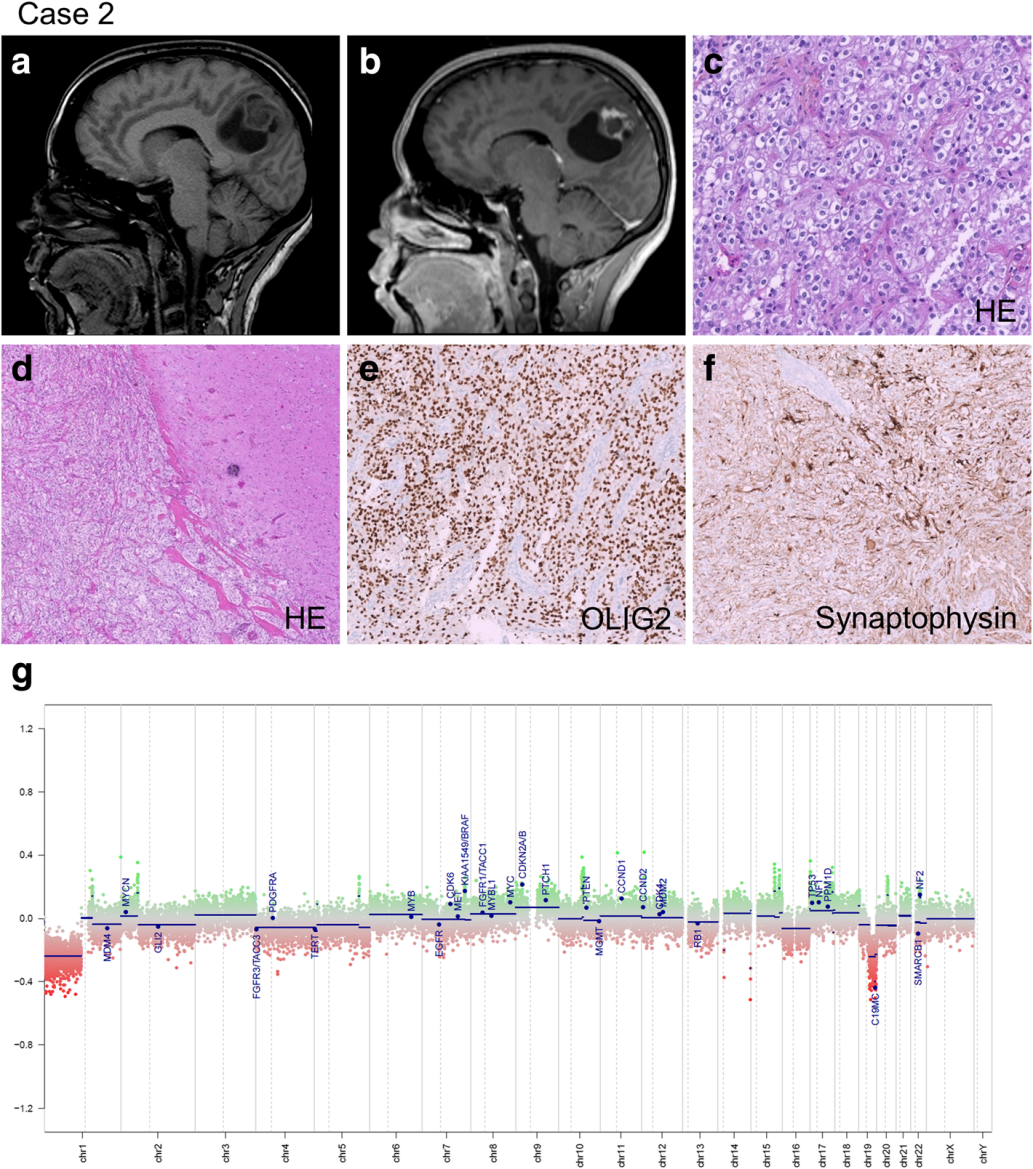
**a, b** MRI showed a cystic tumor with a mural nodule enhanced after gadolinium injection. **c** A proliferation of oligodendrocyte-like cells associated with a rich and branched vasculature. **d** Well demarcation of the tumor from the adjacent cortex. **e, f** The tumor cells strongly expressed OLIG2 and synaptophysin. **g** Copy-number profile derived from DNA methylation arrays revealed 1p/19q codeletion and *KIAA1549:BRAF* fusion

A 31-year-old woman was admitted to La Timone Hospital in March 2012 for epileptic seizures. MRI revealed a parietal cystic tumor with a mural nodule enhanced after gadolinium injection (Fig. 2, a-b). A gross total resection was performed. Pathological examination showed a proliferation of oligodendrocyte-like cells associated with a rich and branched vasculature (Fig. 2, c). Desmoplasia was observed. The tumor was well limited from the adjacent cortex (Fig. 2, d). Granular bodies were evident as well as inflammatory exudates. Ganglionic neurons were lacking. The tumor strongly expressed OLIG2 and synaptophysin but not neurofilament nor chromogranin A (Fig. 2, e-f). No extravascular CD34 expression was observed. KI67 labeling index was 3%. No *BRAF V600E* mutation was recorded by direct sequencing. The diagnosis was “benign glioneuronal tumor compatible with ganglioglioma grade I”. In 2018, this case was included in the series of cases of gangliogliomas lacking *BRAF V600E* mutation in order to search for *EWSR1-PATZ1* mutation and to perform methylation profiling [12]. Methylation profile did not yield a high-confidence score based on the https://www.molecularneuropathology.org classification algorithm, possibly due to a moderate proportion of normal cells / immune infiltration in the tissue sample used for molecular profiling. The copy number profile indicated 1p/19q codeletion and *KIAA1549:BRAF* fusion, as commonly observed in DLGNT (Fig. 2, g). FISH analysis confirmed 1p/19q codeletion, and droplet digital PCR analysis confirmed the *KIAA1549:BRAF* fusion as previously described [13]. The patient did not undergo any adjuvant treatment and after 10 years follow-up, she is still alive with no evidence of relapse nor leptomeningeal spread.

## Discussion and conclusions

In this study we report two cases that fully meet the diagnostic criteria of DLGNTs as proposed by the sixth cIMPACT-NOW update [9], although neither of them presented diffuse growth nor leptomeningeal involvement in spite of a long follow-up. Indeed, both were glioneuronal neoplasm composed of oligodendrocyte-like cells; chromosome arm 1p deletion and a mitogen-activated protein kinase (MAP-Kinase) pathway gene alteration.

**Fig. 3:**
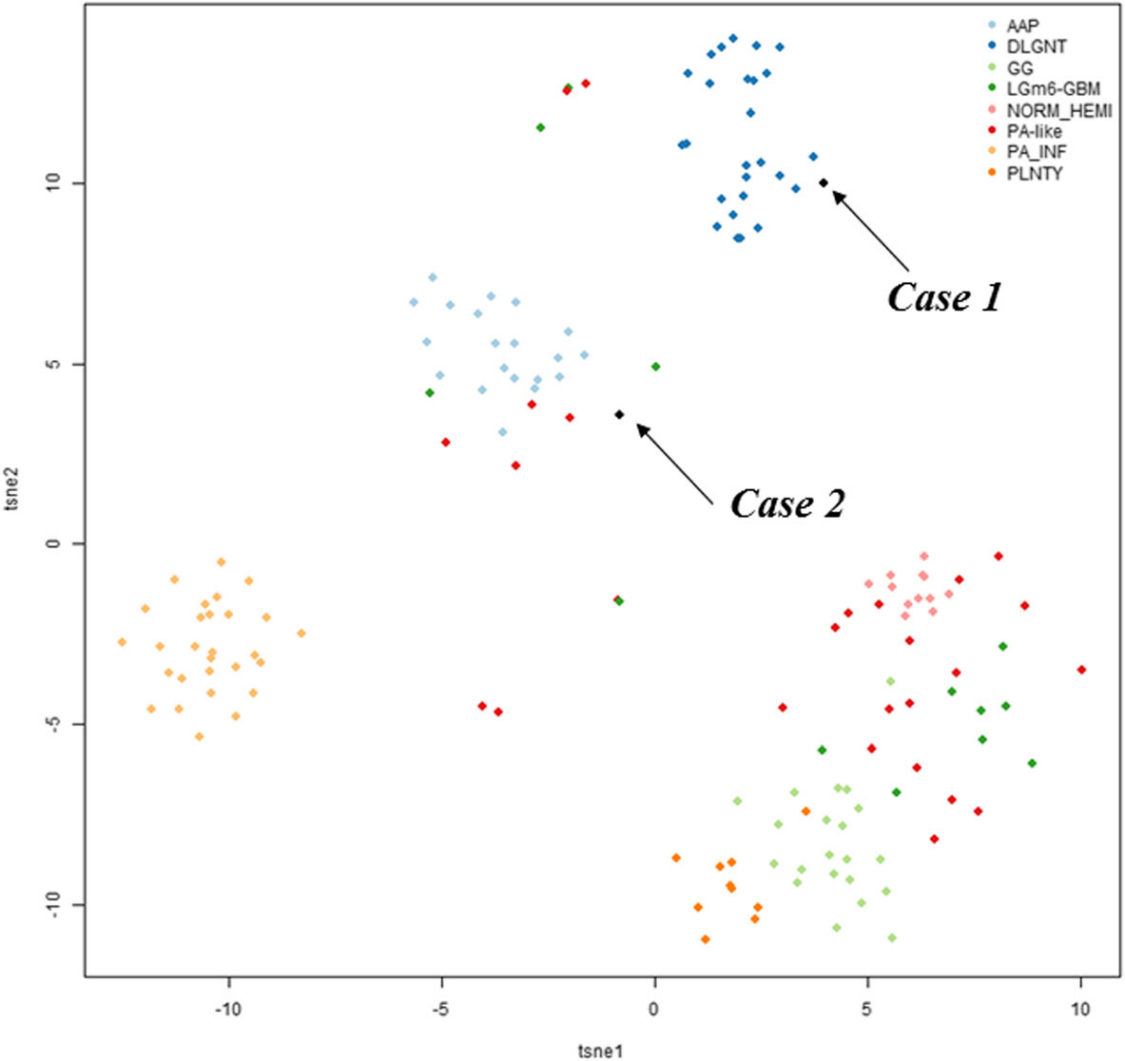
t-SNE plot of the 2 reported cases associated with 156 reference gliomas. Reference classes: AAP, anaplastic astrocytoma with piloid features; DLGNT, diffuse leptomeningeal glioneuronal tumor; GG, ganglioglioma; LGm6-GBM [14]; NORM_HEMI, normal hemisphere; PA-Like [14]; PA_INF, posterior fossa pilocytic astrocytoma and PLNTY, polymorphous low-grade neuroepithelial tumor of the young

In the first case, diagnosis was made on the late relapse, 20 years later, of a tumor initially diagnosed as “diffuse astrocytoma grade II”. In the second case, initial pathological diagnosis was “ganglioglioma grade I” and this tumor was reclassified as DLGNT 8 years later after evidence for both 1p/19q codeletion and *KIAA1549:BRAF* fusion. A t-SNE analysis (t-distributed stochastic neighbor embedding) was performed (Fig. 3) including the following glioma reference classes: ganglioglioma (GG, 21 cases), anaplastic astrocytoma with piloid features (AAP, 20 cases), DLGNT (26 cases), LGm6-GBM (Ceccarelli et al., 2016, 13 cases) [14], PA-like low grade gliomas (PA-like, Ceccarelli et al., 2016, 26 cases), pilocytic astrocytoma (PA_INF, 28 cases), polymorphous low-grade neuroepithelial tumor of the young (PLNTY, 10 cases) and normal hemisphere (Norm_Hemi, 12 cases). Our first case clustered with the DLGNT tumor group, highly supporting its diagnosis. As expected, our second case, which did not demonstrated a significant confidence score on the classification algorithm, possibly due to a moderate proportion of normal cells / immune infiltration in the tissue sample, did not segregated in a clear methylation group. It showed similarity to AAP but was detached from the main cluster of this group. However, in contrast with AAP, this case did not demonstrated features of anaplasia, piloid cytology, *ATRX* loss nor *CDKN2A/B* homozygous deletion. In addition, clinical course was favorable in contrast with the reported evolution of AAP. It was a case of difficult diagnosis, for which pathological and molecular features fully met the diagnostic criteria of DLGNT with no argument towards a differential diagnosis. These two cases point out that the DLGNTs can occur in the absence of leptomeningeal dissemination and in the adult setting, in contrast to initial reports [5]. In such circumstances it is particularly challenging to think of this diagnosis since the pathological features remain non-specific and could mimic pilocytic astrocytoma, ganglioglioma, oligodendroglioma *IDH*-mutant and 1p/19q codeleted, extraventricular neurocytoma, as well as recently reported polymorphous low-grade neuroepithelial tumors of the young (PLNTYs) [15] and tumors possibly related to the last reported as *BRAF* V600E mutant oligodendroglioma-like tumors with chromosomal instability (BRAF-OLT) [16]. All these tumors have in common a focal oligodendrocyte-like proliferation (i.e pilocytic astrocytoma, ganglioglioma) or a more diffuse pattern (oligodendroglioma *IDH*-mutant and 1p/19q codeleted,extraventricular neurocytoma and PLNTYs/BRAF-OLT). Some pathological criteria, when present, might be more characteristic of one entity than others: Rosenthal fibers in pilocytic astrocytoma, lymphocytic cuffing, and eosinophilic granular bodies in ganglioglioma. Ganglion cells, a common feature of ganglioglioma might also be observed in DLGNT and in extraventricular neurocytoma [5, 17]. Extraventricular neurocytoma and oligodendroglioma *IDH*-mutant and 1p/19q codeleted often display a dense branched vasculature. Moreover pilocytic astrocytoma, ganglioglioma, oligodendroglioma *IDH*-mutant and 1p/19q codeleted or extraventricular neurocytoma share with DLGNT the OLIG2 and synaptophysin protein expression whereas PLNTYs are OLIG2-positive but most commonly lack synaptophysin expression [15]. In addition, ganglioglioma and PLNTYs/BRAF-OLTs frequently demonstrate CD34-immunopositivity. Furthermore, as observed in our cases, GFAP expression is commonly lacking in DLGNT whereas it is positive in the differential diagnosis mentioned above [5, 6]. The two cases reported here demonstrated oligodendrocyte-like proliferations of cells co-expressing OLIG2 and synaptophysin associated with a dense, often branched vasculature. Although rare, pathological features of anaplasia such as those observed in our first case (microvascular proliferation, high mitotic count and proliferation index) might occur in DLGNTs. Genetic findings help to distinguish DLGNTs from their main differential diagnoses. DLGNTs share MAP-Kinase alteration with pilocytic astrocytoma, ganglioglioma or PLNTYs/BRAF-OLTs. However, 1p deletion is not a common feature of pilocytic astrocytoma, when present it is usually subclonal [10], and 1p/19q codeletion does not occur. Furthermore, the 1p/19q codeletion is not encountered in ganglioglioma nor in PLNTYs/BRAF-OLTs, but by definition, is the hallmark of oligodendroglioma *IDH*-mutant and 1p/19q codeleted. The diagnosis of our second case was particularly challenging because it was supratentorial, occurring in an adult, and the pathological examination was suggestive of anaplastic oligodendroglioma. However, in spite of a 1p/19q codeletion, *IDH* mutation was lacking and *KIAA1549:BRAF* fusion was present. It is to note that, Badiali et al. [18] previously reported rare cases of gliomas *IDH*-mutant and 1p/19q-codeleted with co-occurrence of *KIAA1549:BRAF* fusion. Therefore, in adults, an oligodendrocyte-like tumor demonstrating 1p/19q codeletion, in the absence of *IDH* mutation should point towards DLGNT and the search for an associated MAP-Kinase alteration should be done by appropriate testing even if the tumor is supratentorial without leptomeningeal dissemination. The first case was also very unusual by the association of 1p/19q codeletion, *PIK3CA* mutation and *BRAF V600E* mutation. As far as we know *PIK3CA* mutation has never been reported. Although MAP-Kinase alteration is always recorded in DLGNT, the most frequent MAP-Kinase alteration reported in DLGNT is the *KIAA1549:BRAF* fusion although *NTRK1/2/3* and *TRIM33:RAF1* fusions might also occur [4]. The *BRAF* V600E mutation seems very infrequent in DLGNT. We could identify only one previously reported case of low-grade glioneuronal tumor with oligodendroglial features and *BRAF* V600E mutation that presented as an isolated temporal lesion that later disseminated. However, 1p data was not available for this case and thus whether it corresponded to a DLGNT remains questionable [19]. Two subtypes of DLGNT have been described by DNA-methylation profiling: DLGNT-MC-1 and DLGNT-MC-2 by Deng et al. [4]. Compared to DLGNT-MC-2, the age at diagnosis was lower in the DLGNT-MC-1 group, (median 5 vs 14 years, *p* < 0.01) and the clinical course less aggressive (5-year OS 100% vs 43% in DLGNT-MC-2). DLGNT-MC-2 is enriched for superimposed 1q gain whereas codeletion of 1p/19q was more frequently observed in DLGNT-MC-1. Our two cases demonstrating 1p/19q codeletion and excellent outcome likely belong to the DLGNT-MC-1 subtype (confirmed in one case, and assumed based on absence of 1q gain in the second).

To conclude, the terminology of “diffuse leptomeningeal glioneuronal tumor” appears unsuitable in our cases which had neither diffuse growth nor leptomeningeal dissemination suggesting that DLGNTs comprises a spectrum of tumors that has yet to be fully clarified. These tumors would be further classified in the large group of “gliomas and glioneuronal tumors driven by MAP-Kinase pathway alterations” in the fifth edition of the WHO classification of CNS tumors.

## Data Availability

Not applicable.

## Abbreviations

AAP: Anaplastic astrocytoma with piloid features
BRAF-OLT: BRAF V600E mutant oligodendroglioma-like tumor
c-IMPACT-NOW: Consortium to inform molecular and practical approaches to CNS Tumor taxonomy – not official WHO
CNS: Central nervous system
CNV: Copy number variation
ddPCR: Droplet digital PCR
DLGNT: Diffuse leptomeningeal glioneuronal tumor
FDG-PET: Positron emission tomography with 2-deoxy-2-fluoro- D-glucose
FISH: Fluorescence in situ hybridization
GG: Ganglioglioma
IDAT files: Intensity data files
LGG: Low grade glioma
MAP-Kinase: Mitogen-activated protein kinases
MRI: *Magnetic resonance imaging*
NGS: *Next-generation sequencing*
PA: Pilocytic astrocytoma
PA-like: PA-like low grade glioma
PLNTY: Polymorphous low-grade neuroepithelial tumor of the young
RENOCLIP-LOC: *Réseau national de neuro-oncologie clinicoPathologique pour les cancers rares du système nerveux central*
t-SNE: t-distributed Stochastic neighbor embedding
WHO: World health organization
